# Klotho Sensitizes Human Lung Cancer Cell Line to Cisplatin via PI3k/Akt Pathway

**DOI:** 10.1371/journal.pone.0057391

**Published:** 2013-02-21

**Authors:** Yan Wang, Lei Chen, Guochang Huang, Dongmei He, Juan He, Wei Xu, Chunying Zou, Feng Zong, Yan Li, Bo Chen, Shuanshuan Wu, Weihong Zhao, Jianqing Wu

**Affiliations:** 1 Department of Geriatrics, The First Affiliated Hospital of Nanjing Medical University, Nanjing, China; 2 Department of Surgery, Memorial Sloan-Kettering Cancer Center, New York, New York, United States of America; 3 Department of Orthodontics, Nanjing Stomatological Hospital, Affiliated to Medical School, Nanjing University, Nanjing, China; Virginia Commonwealth University, United States of America

## Abstract

Klotho was first identified in 1997 and has been considered as an anti-aging gene. Emerging evidence demonstrates that klotho has a close relationship with cancers, including lung cancer, breast cancer, etc, by inhibiting the proliferation and promoting apoptosis of cancer cells. Cisplatin has been the most widely used drug in the first-line chemotherapy. However, the increase in cisplatin-resistant cancer cells has become a major obstacle in clinical management of cancers. In our study, we for the first time demonstrated that klotho could attenuate the resistance of lung cancer to cisplatin based chemotherapy and the apoptosis of the resistant cells with klotho overexpression was markedly increased. However, klotho knockdown cells showed enhanced resistance to chemotherapy. Further analysis showed that inhibition of PI3K/Akt pathway with specific inhibitor (LY294002) attenuated the promotive effects on cancer growth following interfering with klotho shRNA. Moreover, we demonstrated that klotho modulated the resistance to cisplatin in a xenograft nude mice model. These observations suggested that klotho could improve the resistance of lung cancer cells to chemotherapy and may serve as a potential target for the gene therapy of lung cancers resistant to cisplatin based chemotherapy.

## Introduction

Non-small cell lung cancer (NSCLC) accounts for 75–85% of lung cancer cases and chemotherapy plays an important role in the treatment of lung cancer [1]. Cisplatin has been the most widely used drug in the first-line chemotherapy. Cisplatin can activate several signaling pathways, including those involving ATR, p53, p73, and MAPK, and activate apoptosis [2], [3]. Its cytotoxicity is attributed to the formation of DNA adducts, which cause inter- and intra-strand cross-linking which inhibits DNA replication. However, the resistance of lung cancer to chemotherapy has been a major factor affecting the therapeutic efficacy in the treatment of lung cancer. Thus, it is imperative to develop strategies to improve the resistance of human lung cancer to platin based chemotherapy. The mechanism underlying the resistance of cancer cells to chemotherapy is complicated and involves the activation of PI3K/Akt (also known as PI3K/PKB) pathway, the loss of p53 function, over-expression of HER-2/neu and anti-apoptotic bcl-2, and the compromised caspase activation [2], [3]. Thus, to deeply investigate the mechanism underlying the resistance of cancer cells to chemotherapy has great clinical importance in the treatment of cancers.

Klotho is a newly found anti-aging gene and was originally identified in klotho homozygous mutant mice (kl-/-) which showed a human-like aging-related syndrome and develop multiple disorders such as hypogonadism, ectopic calcification, osteoporosis, skin atrophy, and pulmonary emphysema [4]. However, mice with klotho over-expression have an extended life span that is 30% longer in males and 20% longer in females [5]. Klotho gene encodes a single-pass type-1 transmembrane or secreted form of klotho protein through alternative RNA splicing [6]. Klotho was shown to exert an inhibitory effect on the insulin-like growth factor-1 (IGF-1) pathway both in human beast cancer cells and pancreatic cancer cells [7], [8]. Our previous study also found this phenomenon in human lung cancer cells (A549 cells) in which klotho also conferred a pro-apoptotic effect through the bax/bcl-2 pathway [9]. PI3K/Akt pathway is one of the important down-streams of IGF-1 pathway, and numerous studies have confirmed its role in the apoptosis of cancer cells [10]–[12], and could sensitisize these cancer cells to cisplatin.

In this study, we hypothesized klotho could inhibit the PI3K/Akt pathway and further to alleviate the resistance of lung cancer cells to cisplatin and may serve a potent candidate for the gene therapy of lung cancer.

## Materials and Methods

### Ethics statement

This study was carried out in strict accordance with the recommendations in the Guide for the Care and Use of Laboratory Animals of the National Institutes of Health. The protocol was approved by the Committee on the Ethics of Animal Experiments of Nanjing medical university (Permit Number: 2120474). All surgery was performed under sodium pentobarbital anesthesia, and all efforts were made to minimize suffering.

### Cell culture and transfection

Human lung cancer cell line (A549 cells) was obtained from the American Type Culture Collection (ATCC). The H460 cells and cisplatin-resistant A549 and H460 (A549DDP and H460DDP) cells were kindly provided by the Prof. Zhou in Shanghai Pulmonary Hospital. All cell lines were maintained in RPMI 1640 (Life Technologies, Inc., Gaithersburg, MD) containing 10% fetal bovine serum (FBS) (Life Technologies, Inc.), 100 U/mL penicillin, 100 U/mL streptomycin, 2 mM glutamine in a humidified atmosphere with 5% CO_2_ at 37 °C. To maintain drug resistance, A549/DDP and H460/DDP cells were grown in RPMI 1640 containing 2 µg/ml DDP and then in DDP free RPMI 1640 two days before experiments. A549DDP cells reaching 80% confluence were transfected with pCMV6-MYC-KL and its specific shRNAs in the presence of lipofectamine 2000 (Invitrogen). The shRNAs were as follows:

sh-1: CCTAAGCTCTCACTGGATCAATCCTCGAA;

sh-2: CTGAGGCAACTGCTTTCCTGGATTGACCT;

sh-3: GGTCACTCACTACCGCTTCTCCATCTCGT;

sh-4: GTTACAGCATCAGGCGTGGACTCTTCTAT;

All plasmids were purchased from the OriGene (Rockville, MD, USA).

### MTT Assay

The half maximal inhibitory concentration (IC50) of A549DDP and A549 cells were determined by the MTT (3-[4,5-dimethylthiazol-2-yl]-2,5-diphenyltetrazolium bromide) assay (Sigma). Cancer cells were seeded into 96-well plates (1×10^4^ cells per well), and treated with cisplatin at different concentrations for 48 h. After incubation, the media was replaced with 50 µL of MTT reagent (2 mg/mL) followed by further incubation in an atmosphere with 5% CO_2_ at 37 °C for 2 h. Then, the media were removed and dimethylsulfoxide (DMSO) (150 µL) was added to each well. The optical density (OD) of each well was measured using a microplate reader at 560 nm.

### Morphologic examination

Twenty-four hours after transfection, the cells were treated with 80 µM (IC50 for A549DDP) cisplatin for 8 h, and 60 µM for H460DDP, and then washed once in phosphate buffer saline (PBS) followed by fixation in cold methonal: acetone (1:1) for 5 min. After washing thrice in PBS for 5 min, these cells were treated with 4 µg/ml 4′, 6-diamidine-2′-phenylindole dihydrochloride (DAPI) (Sigma) for 10 min at room temperature. Cells were then examined by fluorescence microscopy. Cells were randomly selected for examination at a high magnification (×400) and photographed. Both normal cells (big nuclear, dispersion and homogeneous fluorescence) and the apoptotic cells (nuclear shrinkage and hyperchromatic nuclei) were counted under each field.

### Flow cytometry

Cells were harvested and gently disaggregated to a single cell suspension. Staining was made according to the manufacturer's protocol. The rate of apoptosis induced by anticancer regimens was analyzed by flow cytometry using an annexin V-FITC/PI kit (BD Biosciences, San Diego, CA) following the manufacturer's instructions.

### Real-time RT-PCR

Total RNA was extracted from cells with TRIzol reagent (Invitrogen, San Diego, CA) according to the manufacturer's instructions, and quantified by measuring the absorbance at 260 nm. Gene expressions were detected by quantitative real-time RT-PCR (qRT-PCR) using the standard SYBR Green RT-PCR kit (Takara, Dalian, China) according to the manufacture's instructions. The cDNA was synthesized using the RevertAid First-Strand cDNA Synthesis kit (Fermentas, Vilnius, Lithuania) according to the manufacturer's instructions. Primer sequences were designed with Origene Technologies, Inc. The primers used were as follows:

*klotho* sense: 5′-GCTCTCAAAGCCCACATACTG -3′;

antisense: 5′-GCAGCATAACGATAGAGGCC -3′;

*β-actin* sense: 5′-TGACGTGGACATCCGCAAAG-3′;

antisense: 5′-CTGGAAGGTGGACAGCGAGG-3′;

PCR conditions consisted of pre-denaturation at 94 °C for 5 min, followed by 30 cycles of denaturation at at 94 °C for 30 sec, annealing at 60 °C for 30 sec and extension at 72 °C for 30 sec and a final extension at 72 °C for 10 min.

### Western blot assay

24 h and 48 h after transfection, cells were washed twice in ice-cold PBS and proteins were extracted from cells by lysis in RIPA buffer (150 mM NaCl, 1% [v/v] NP-40, 0.5% [w/v] sodium deoxycholate, 0.1% [w/v] sodium dodecyl sulfate (SDS), 50 mM Tris HCl [pH 8], 10 mM EDTA, and 1 mM PMSF [Sigma]) for 30 min at 4 °C and subsequent centrifugation for 15 min at 12,000 g. Protein concentration was determined with a BCA kit (Pierce) according to the manufacturer's instructions. Then, 60 µg of protein was loaded onto a 10% (w/v) SDS-polyacrylamide gel, electrophoresed, and transferred onto a PVDF membrane (Millipore Corporation Billerica,MA 01821) which was then blocked for 2 h at room temperature with blocking buffer (TBS containing 0.1% [v/v] Tween 20 [Sigma] and 5% [w/v] milk powder). Primary antibodies (applied for 1 h at room temperature, or overnight at 4 °C) were: anti-phospho-akt,anti-akt, (Cell Signaling Technology, Inc., Beverly, MA), anti-Bax,anti-klotho, and anti-GAPDH (Santa Cruz). Antibodies were diluted to 1:1,000, except for the anti-GAPDH antibody (1:500). Thereafter, membranes were incubated for 2 h with HRP-conjugated secondary antibodies (Vector Laboratories, Inc., Burlingame, CA) (horse anti-mouse or goat anti-rabbit; 1:20000). Visualization was done by using an ECL kit, and the signals were quantified by scanning densitometry.

### Transduction of Lentivirus

The lentiviral vectors expressing short hairpin RNAs (shRNAs) were successfully constructed by OriGene (Rockville, MD, USA) as previously described. The effectively constructed klotho-shRNA oligonucleotide sequences were as follows, sense: 5′-*CGCGTCCCC*CTGAGGCAACTGCTTTCCTGGATTGACCT*TCAAGAGAGGTCAATCCAGGAAAGCAGTTGCCTCAGTTTTTGGAAAT*-3′.

The sequences were inserted into the *mul*I and *cla*I enzyme sites of pLVTHM vector, respectively. For recombination reaction using pCMV-dR8.74 and pCMV-VSV-G vectors (*Addgene*, Cambridge, MA), lentiviral vector DNAs and packaging vectors were then transfected into 293T cells. After 48 hr transfection, supernatants containing lentiviruses were harvested, centrifuged at 1800 g for 10 min, and filtered through a 0.45 µm poly (vinylidene difluoride) Durapore membrane (Millipore, Billerica,MA, USA) to remove any nonadherent packaging cells. Resistant lung cancer cell line (A549DDP) was infected with the lentivirus. Then, the infected cells were selected by puromycin (0.4 µg/ml) for 14 days. A549DDP cells infected with lentivirus-mediated shRNA targeting klotho (sh-klotho) or lentivirus-mediated shRNA (scramble) were named A549DDP-lenti-sh-2 or A549DDP-lenti-scramble, respectively.

### In vivo experiments in nude mice

For in vivo experiments, Male athymic nude mice (BALB/c nu/nu, four weeks old) were used. They were injected subcutaneously with A549DDP-lenti-sh-2 or A549DDP-sh-scramble cells. When tumors measured an average volume of 100 mm3, the mice (6 per group) were treated with cisplatin (3.5 mg/kg, 2 times a week) or PBS for 21 days. Tumors were measured with calipers. Tumor volumes were calculated by the following formula: tumor volume (mm3)  =  0.5*length (mm)*width (mm)*width(mm).

### Statistical analysis

Data were expressed as mean ± standard deviation (SD). Experiments were performed at least in triplicate. Comparisons were done with two-tailed Student's t test or ANOVA. A value of *P*<0.05 was considered statistically significant.

## Results

### Klotho expression was down-regulated in cisplatin-resitant cells

**Figure 1:**
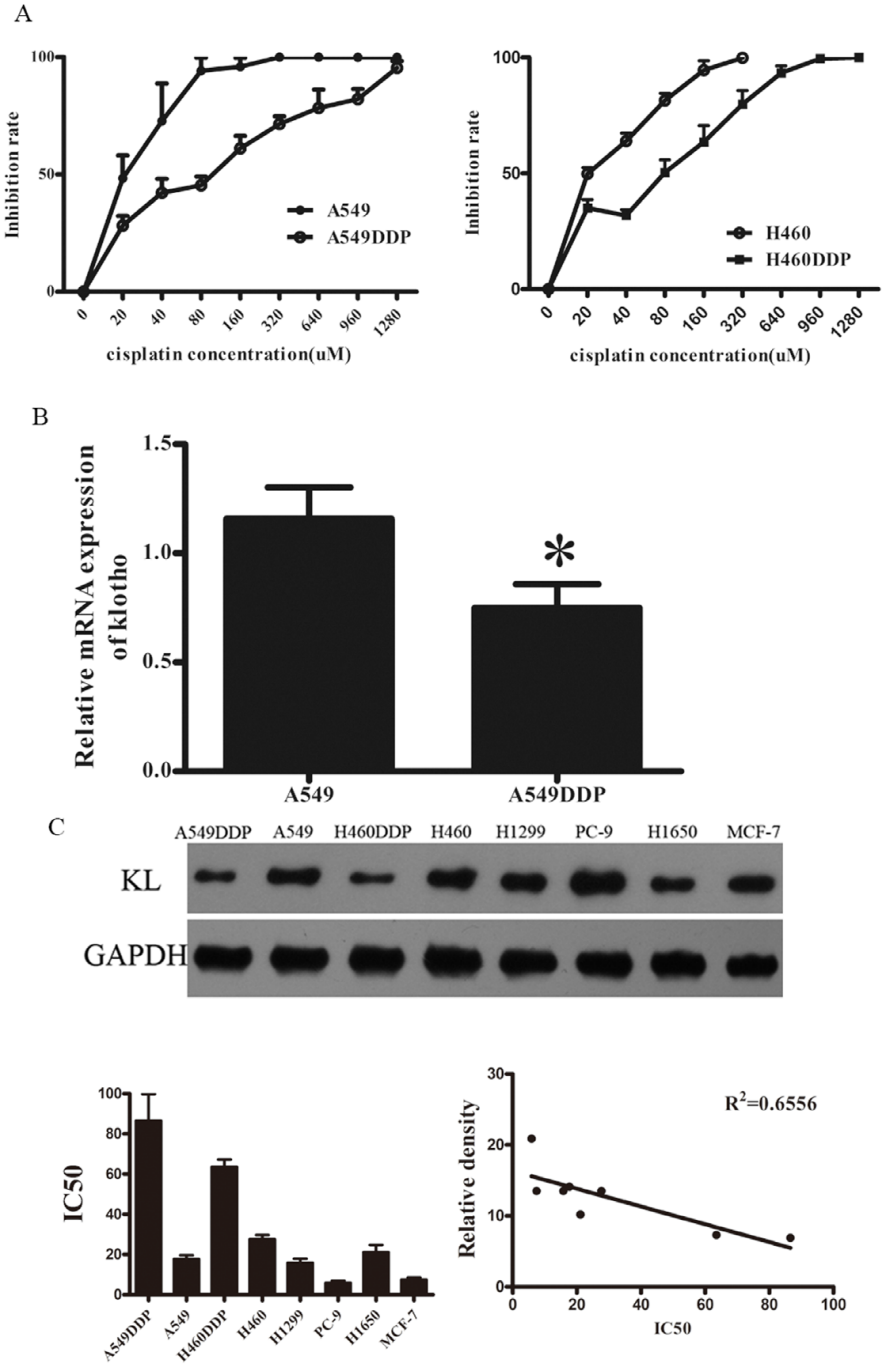
IC50 and klotho expression in cisplatin resistant cells and their parent cells. **A**: Two cell lines treated with cisplatin showed difference in IC50. **B**: Quantitative RT-PCR for the detection of klotho expression in A549 cells and A549DDP cells. **C**: Western blot analysis of klotho expression in a panel of lung cancer cell lines including two cisplatin resistant variants and a breast cancer cell line (MCF-7). Klotho expression is higher in parental H460, and A549 cell lines as compared to the cisplatin resistant variants. Higher Klotho expression was also detected in H1299, PC-9, H1650 and MCF-7 cell lines. GAPDH served as an internal reference. Detection was performed in triplicate and data were presented as mean±SD (*P<0.05)

To confirm the resistance of A549DDP and H460DDP cells, MTT assay was done to measure the IC50 of A549 cells and A549DDP cells. Results showed the IC50 was 17.7±1.93 µM in A549 cells and 86.6±13.2 µM in A549DDP cells,while H460DDP and its parent cell line were 63.5±3.8 and 27.7±2.0 respectively (Fig 1A). The expression of klotho was determined by real-time PCR and western blot assay. The relative mRNA level of klotho in A549DDP cells was about 35% lower than that in A549 cells (Fig 1B). Western blot assay showed the same trend in the protein expression of klotho in both cell lines (Fig 1C). Furthermore, we identified the expression of klotho and IC50s in other cell lines, including H1299, PC-9, H1650 and MCF-7 (Fig 1C), and we found that the endogenous klotho expressions were negatively related to cisplatin sensitivity (Fig 1C), and statistical analysis demonstrated an inverse relationship between klotho expressions and IC50s. Thus, we speculated that there was relationship between klotho expression and the resistance to cisplatin and the klotho expression might contribute to the resistance.

### Over-expression of klotho could inhibit Akt signal pathway and induce apoptosis of A549/DDP cells

**Figure 2:**
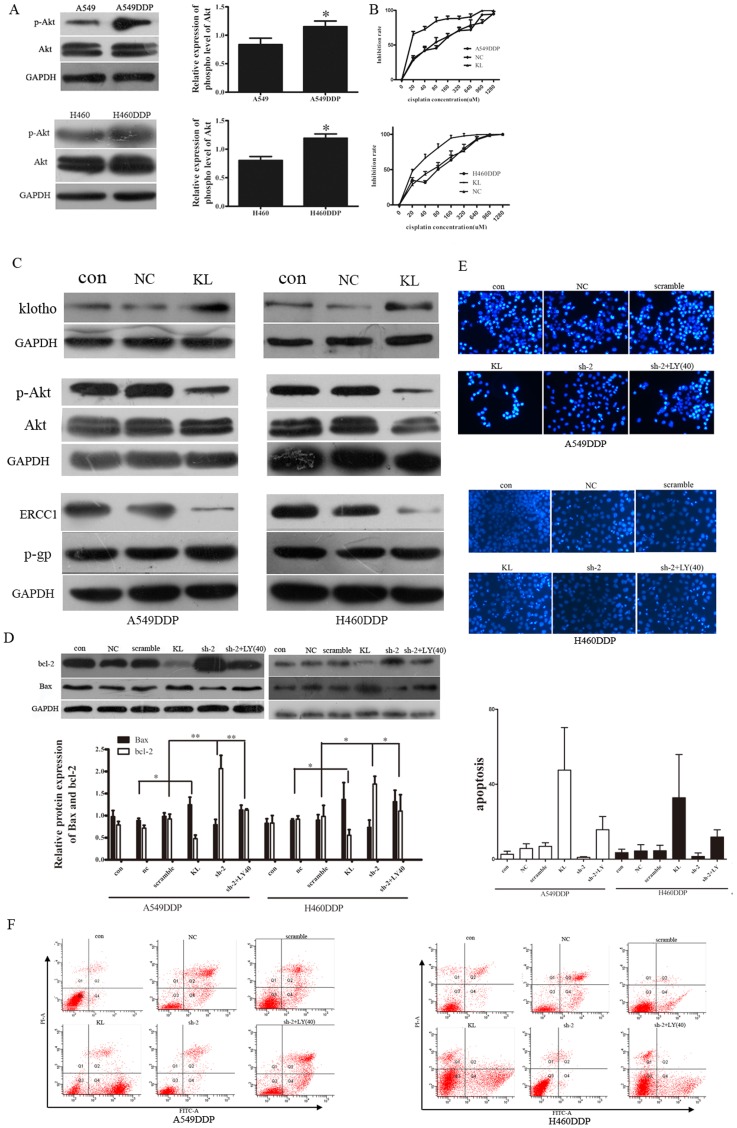
Klotho transfection reduced IC50 of both cell lines and inhibited Akt signal pathway. **A, B:** Western blot assay showed the p-Akt expression was enhanced significantly in the cells resistant to cisplatin. **C**: MTT assay showed a great decrease in IC50 of klotho transfected cells. **D**: Western blot assay showed the elevated expression of klotho in cisplatin resistant cells. P-Akt expression was down-regulated in cisplatin resistant cells after klotho transfection. GAPDH served as an internal reference. Experiment was performed in triplicate and data were presented as mean±SD (*P<0.05)

To determine the role of klotho in the cisplatin-resistance of cisplatin resistant cells, we first detected the p-AKT expression in A549, H460 and their cisplatin resistant cells. Results demonstrated that the expression of phosphorylated Akt was significantly lowered in the A549 and H460 cells (Fig 2A). Then, cancer cells with klotho over-expression were prepared. As shown in Figure 2C, the protein expressions of klotho in both cell lines were markedly increased after transfection. The effect of klotho over-expression on the resistance of A549/DDP and H460/DDP cells to cisplatin was determined by MTT assay. Data are shown in Figure 2B. The proliferation of cells transfected with klotho was significantly suppressed when compared with the control group. The mean IC50s were lower in klotho transfected cells implying that these cells were more sensitive to cisplatin. Moreover, the expression of phosphorylated Akt was negatively related to the klotho expression, cells with klotho over-expression showed a decreased expression of phosphorylated Akt when compared with the control group (Fig 2C). Then, we detected the apoptosis of cells undergoing klotho transfection. DAPI staining is a method to detect apoptosis. Apoptotic cells will morphologically present the features of apoptosis following DAPI staining: bright nuclear condensation and perinuclear apoptotic bodies. Flow cytometry is a technique for counting, examining and sorting microscopic particles suspended in a stream of fluid. It allows simultaneous multiparametric analysis of the physical or chemical characteristics of single cells flowing through an electronic detection apparatus, and it is commonly used for detecting apoptosis. Western blot assay revealed that the expression of Bax and bcl-2 had the same trends to those in flow cytometry and DAPI staining (Fig 2D). The DAPI staining showed the number of apoptotic cells with klotho over-expression was dramatically increased when compared with those undergoing transfection with blank plasmid, but the apoptosis of cells treated with blank plasmid was comparable to that in the control (Fig 2E). Flow cytometry analysis showed the apoptosis of the A549DDP cells transfected with klotho was 47%, while H460DDP was 19.8%, which means they were more sensitive to cisplatin compared to the negative controls (Fig 2F). These findings suggested that klotho could increase the sensitivity of cisplatin resistant cells to cisplatin by elevating the apoptosis in a Akt dependent manner.

Further, we investigated the phenotype of the drug-resistant cells and the parent cells, and we found that ERCC1 was down-regulated in the klotho transfected cells, while the p-gp, a gene related to the drug efflux, and contributed to the most cisplatin resistance, was not affected by klotho (Fig 2C). These results implied that klotho may affect the repair of the cells through ERCC1 to alter the resistance of the cancer cells rather than increase energy-dependent efflux of hydrophobic drug.

### Klotho knockdown inhibited apoptosis and increased resistance to cisplatin in A549/DDP cells

**Figure 3:**
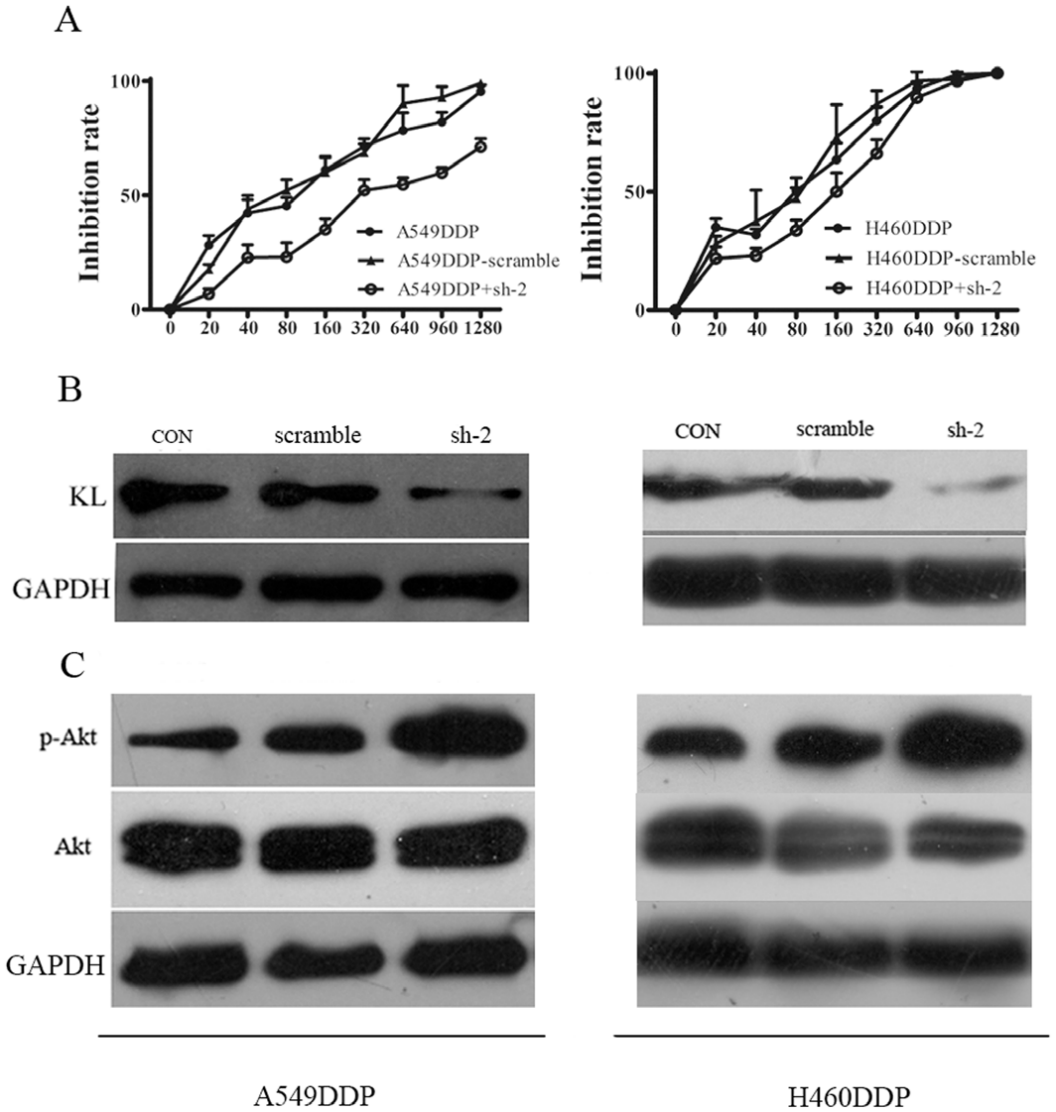
Klotho knockdown with specific shRNA increased IC50 and p-Akt expression in cisplatin resistant cells. **A:** The ERCC1 and p-gp protein levels in cisplatin resistant cells were identified by western blot. **B**: A549DDP and H460DDP cells were transfected with klotho specific shRNA or scrambled RNA as a negative control. MTT assay showed an increased IC50 of cells transfected with shRNA. **C**: Western blot assay confirmed the klotho expression was down-regulated in the cells following transfection with shRNA. **D**: The p-Akt was also detected by western blot assay. Results showed the p-Akt expression was up-regulated significantly following shRNA tranfection. GAPDH served as an internal reference. Experiment was performed in triplicate and data wer presented as mean±SD (*P<0.05).

To further investigate the role of klotho in the resistance of the cells to chemotherapy, klotho knockdown was performed in A549DDP and H460DDP cells with specific shRNAs. Four shRNAs were designed and transfected into A549DDP or H460DDP cells as described in our previous study in which results demonstrated that the sh-2 showed the best interference efficiency [9]. In this study, sh-2 was employed in the following experiments. As shown in Figure 3B, western blot assay demonstrated the expression of klotho was significantly down-regulated by about 50% as compared to the negative control group in both cell lines. We subsequently explored the effect of klotho knockdown on the sensitivity of the resistant cells to cisplatin in which MTT assay was employed to examine the IC50 of A549DDP and H460DDP cells (Fig 3A). Results showed the IC50 of shRNA transfected cells had a pronounced increase in the IC50 as compared to the negative control group. Especially in A549DDP cells, the mean IC50 of cells transfected shRNA was 452.5±20.69 µM (Fig 3A).

Furthermore, the expression of total-Akt, phospho-Akt (p-Akt), Bax and bcl-2 were examined in klotho shRNA transfected cells. Western blot assay showed that the klotho knockdown led to the significant increase in the expression of phospho-Akt (Fig 3C) and bcl-2, but the pronounced decrease in the expression of Bax (a pro-apoptotic protein) (Fig 2D). DAPI staining revealed the apoptosis of cells undergoing shRNA transfection was markedly reduced demonstrated by microscopy as compared to cells receiving transfection with scramble shRNA (Fig 2E). Flow cytometry showed the apoptosis of the cells was 0.2% in A549DDP and 0.1% in H460DDP compared to cells transfected with scramble (Fig 2F). These findings demonstrated that klotho down-regulation decreased the apoptosis via leading to the imbalance in the expression of apoptosis related proteins (Bax and bcl-2).

### Increased resistance following klotho down-regulation was attenuated by Akt inhibition with LY294002

**Figure 4:**
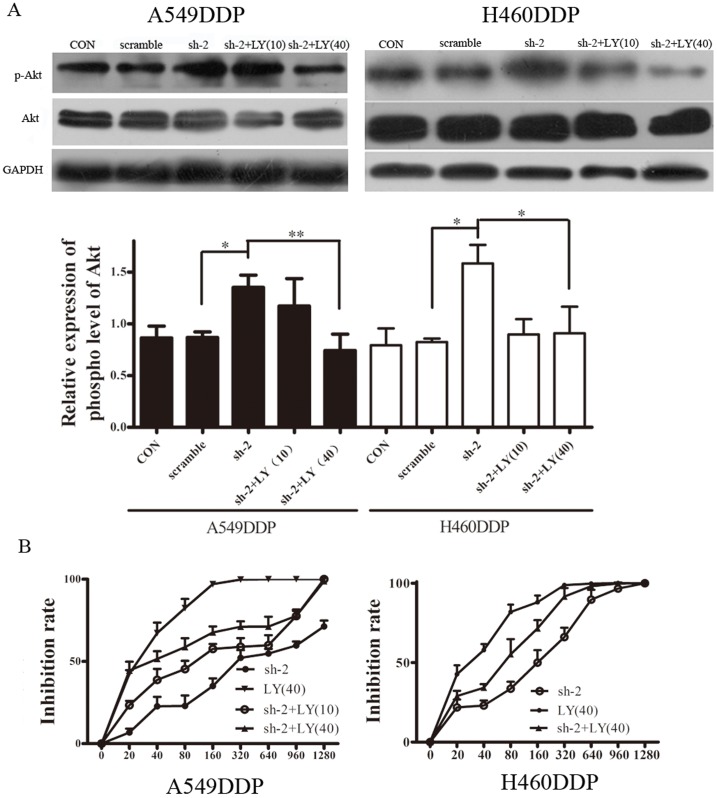
Inhibition of PI3K/Akt signal pathway increased chemotherapeutic sensitivity and promoted apoptosis of shRNA transfected cisplatin resitant cells. A: A549DDP and H460DDP cells transfected with shRNA were treated with LY294002 (10 and 40 µM). Western blot assay demonstrated that LY294002 could significantly lower the p-Akt expression, especially after treatment with 40 µM LY294002. B: Cells transfected with shRNA were treated with LY294002 at different concentrations, and MTT assay was performed to measure the IC50. C: Apoptosis related proteins, Bax and bcl-2, were detected. Results showed klotho over-expression could increase the Bax/bcl-2 ratio while the klotho knockdown decreased it. LY294002 could reverse the decreased ratio following shRNA transfection. GAPDH served as an internal reference. Experiments were performed in triplicate and data presented as mean ± SD (* P<0.05, ** P<0.01).

To further explore whether klotho could improve the resistance of the cisplatin resistant cells to cispaltin through regulating the p-Akt expression, cells transfected with klotho specific shRNA were treated with 10 µM and 40 µM LY294002, a phosphoinositide-3 kinase inhibitor, to elucidate the role of Akt in the increased resistance to chemotherapy following klotho knockdown. LY294002 significantly inhibited the expression of p-Akt in the shRNA transfected cells, especially after treatment with 40 µM LY294002 (Fig 4A), as compared to the negative control group. Then, MTT assay was used to determine the sensitivity of these cells to cisplatin. As shown in Fig 4B, the IC50 of the A549DDP cells treated with LY294002 at 10 µM and 40 µM was 92.97±10.04 µM and 50.52±5.63 µM, respectively, which were markedly lower than that in the control group (452.5±20.69 µM; P<0.01), and the same changes were seen in the H460DDP cells. Therefore, 40 µM LY294002 was used in the following experiments. As shown in Fig 2D, Bax, a pro-apoptotic protein, was significantly up-regulated in cells undergoing LY294002 treatment as compared to cells without treatment with PI3K/Akt inhibitor. On the contrary, the expression of bcl-2 was significantly down-regulated after LY294002 treatment. Moreover, DAPI staining and flow cytometry (Fig 2E, F) showed the same results. These findings suggested that klotho could alleviate the resistance of cancer cells to chemotherapeutics by regulating the apoptosis in a PI3K/Akt signal pathway dependent manner.

### Klotho knockdown *in vivo* significantly increased the resistance of the lung cancer cells to cisplatin

**Figure 5:**
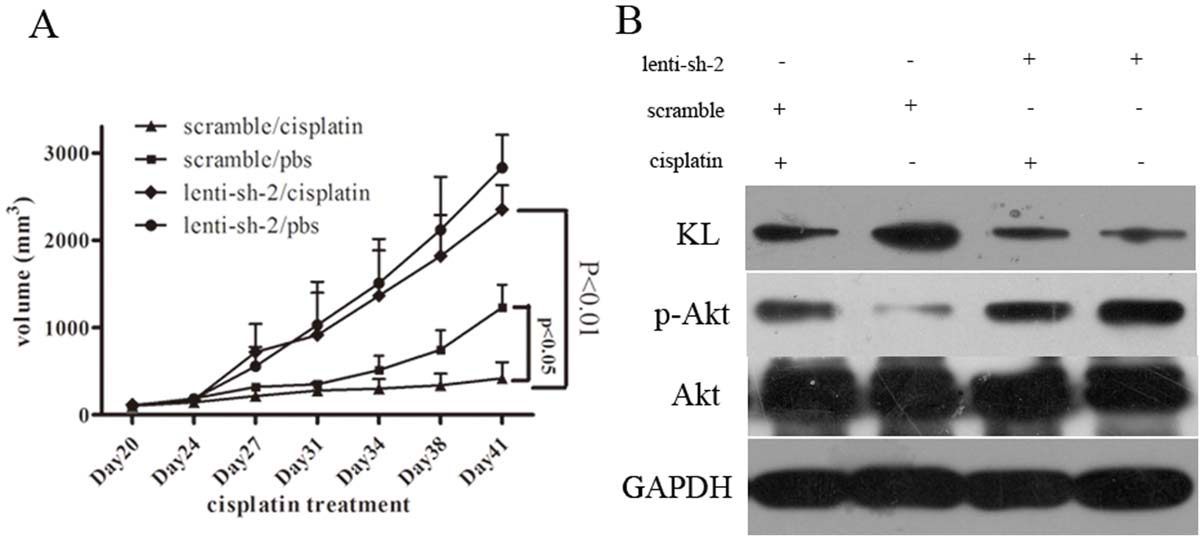
The apoptosis of the cells and the *in vivo* experiment. **A:** Apoptosis of the cells analyzed by Flow cytometry. **B:** Apoptosis of the cells was conducted by DAPI staining. Condensed chromatin was examined by a fluorescence microscopy. **C**: *In vivo* chemoresistance assay of klotho knockdown cells. Xenograft tumor volumes in mice treated with cisplatin or physiological saline are shown. Tumor volume is presented as the mean ± SD. **D**: Expression of klotho and p-Akt in tumor tissue from the mice.

Based on the *in vitro* experiment of klotho in the cisplatin resistance of lung cancers, we further examined if klotho expression affects the cispaltin sensitivity *in vivo*, A549DDP-lenti-sh-2 and A549DDP-lenti-scramble cells were injected subcutaneously into the right flank of nude mice. Cisplatin could significantly inhibit the tumor growth in mice injected with A549DDP-lenti-scramble cells compared to the phosphate buffer control, however, mice injected with A549DDP-lenti-sh-2 cells showed no significantly inhibition compared to the control group (Fig 5A). Western blot further indicated that the expression of klotho in A549DDP-lenti-sh-2 solid tumors was weak, while it was positive in the scramble control solid tumor. Similar results with the *in vitro* studies were identified in the expression of p-Akt (Fig 5B). These findings suggest that klotho contributes to cisplatin resistance in lung cancer cells in xenograft tumor models, and PI3K/Akt was involved in the procedure.

## Discussion

The resistance of cancer cells to chemotherapy has been one of the major challenges in the clinical treatment of cancers. In our previous study, results demonstrated that the new anti-aging gene klotho was crucial for the proliferation and apoptosis of lung cancer cells (A549 cells), mainly through inhibiting the IGF-1/R pathway [9]. Akt, a serine/threonine kinase, plays an important role in oncogenesis [13]–[15], and altered expression of Akt has been observed in various human cancers [16]–[18]. Akt is also a key downstream protein of IGF-1/R pathway. Increasing evidence supports that Akt is involved in the resistance of cancer cells to chemotherapy and radiotherapy [18], [19]. Recently, several studies have identified that inhibition of Akt activation can reduce the cell survival and improve the cisplatin resistance [20], [21]. It is reported that Akt is involved in cisplatin resistant in several cancers [22]–[24]. Once activated PKB/Akt is phosphorylates and actives target proteins involved in many different cellular functions, which span cell cycle progression, cell survival, ribosome biogenesis, protein translation and cell motility 25–27. Klotho was first found in 1997 as a putative aging-suppressor gene in mice that extended life span when overexpressed and induced a premature aging syndrome when disrupted [5]. Since the klotho can inhibit the IGF-1/R signaling pathway in our previous study, and autophosphorylation of IGF-1R induces the activation of Akt by phosphorylation, we hypothesized that klotho could alleviate the resistance of lung cancer cells (A549DDP and H460DDP cells) to chemotherapeutics in which Akt plays an important role.

In the present study, cisplatin resistant NSCLC cells (A549DDP and H460DDP cells) and its parent cells (A549 and H460 cells) were employed for experiments. First, the klotho expression was measured in both cell lines, and results showed the klotho expression at both mRNA and protein levels in the resistant cells was significantly lowered as compared to the parent cells (P<0.05). However, the expression of p-Akt in the cisplatin resistant cells was significantly higher than that in the parent cells. In addition, the high IC50s were significantly reduced in A549DDP and H460DDP cells after klotho up-regulation. However, klotho down-regulation by transfection with specific shRNA increased the resistance to cisplatin, accompanied by an increase in p-Akt expression. Previous studies have identified Akt as an important protein in the IGF-1 mediated cell survival [28]. Constitutively, Akt activation prevents from the extracellular matrix induced apoptosis [29]. There are 2 major mechanisms of drug resistance in cells: first, various changes in cells that affect the capacity of cytotoxic drugs to kill cells, including alterations in cell cycle, enhanced DNA repair activity, defective apoptosis pathway, altered metabolism of drugs, etc; second, increased energy-dependent efflux of hydrophobic drugs, represented by overexpression of a family of energy-dependent transporters, known as ATP-binding cassette transporters, such as P-glycoprotein (P-gp)[30]. ERCC1 (excision repair cross-complementation group 1) is a limiting factor in nucleotide excision repair, removes DNA adducts and repairs DNA double-strand breaks [31], [32]. High levels of ERCC1 are correlated with resistance to cisplatin-based chemotherapy in patients with NSCLC [33], [34]. P-gp was the first discovered human ABC transporter, and was encoded by the MDR1 gene[35]. P-gp is one of the most clinically important transmembrane transporters and plays an important role in drug resistance. To identify the underlying mechanism of the resistance of our cells, we further explored the ERCC1 and p-gp proteins in the transfected cells, and we found that ERCC1 was significantly decreased in the klotho transfected cells compared to the negative controls, however, no significant differences were seen in the expression of p-gp. These findings demonstrated that klotho influenced the resistance of lung cancer cells to cisplatin, which was related to the PI3K/Akt signaling pathway. Further, klotho could modulate the repair activity of the DNA through regulation of ERCC1, then change the resistance of the cancer cells. In the present study, to deeply investigate the role of Akt in the resistance of the cells, we found that Akt inhibition by its specific inhibitor (LY294002) reduced the elevated IC50 following klotho shRNA transfection accompanied by reduction of p-Akt expression. To identify the results *in vitro*, we performed an *in vivo* experiment in nude mice. We found that the decreased antitumor efficacy *in vivo* was associated with the inhibition of klotho expression and the activated PI3K/Akt pathway. These results confirmed that klotho, an anti-aging gene, could alleviate resistance of A549DDP and H460DDP to cisplatin by inhibiting Akt signal pathway.

The regulation of apoptosis is complicated and a number of genes are involved in this process, including Bax/bcl-2, cell-regulatory proteins, etc [36]. Our study showed the ratio of Bax/bcl-2 was increased dramatically after klotho over-expression in cisplatin resistant cells, while the klotho knockdown decreased this ratio accompanied by inhibition of Akt signal pathway. This was consistent with the trend of p-Akt expression and IC50. We detected the apoptosis of the cells, and our results demonstrated klotho played a key role in the apoptosis of chemotherapy resistant cells: increase in klotho expression was closely related to more apoptotic. Based on the results above, we speculate that klotho plays a key role in the regulation of Bax/bcl-2 ratio, and can alleviate the resistance of A549DDP and H460DDP cells to chemotherapy by promoting the apoptosis. The resistance of the lung cancer cells to chemotherapeutics is related to the low klotho expression. Several studies have identified that methylation of klotho gene is a major cause of its down-regulation in cervical cancinoma [37],breast cancer [38] and colorectal cancer [39], and whether this is true in lung cancer cells remains to be investigated in future studies.

In summary, our results demonstrate that the klotho, an anti-aging gene, is associated with the resistance of lung cancer cells to cisplatin and may alleviate the resistance mainly through modulating the PI3K/Akt signal pathway and the expression of bax/bcl-2. Moreover, our results may have important clinical implications in the chemotherapy of lung cancer because the therapeutic efficacy is often impeded by the presence of cisplatin resistance. However, our results are derived from in vitro experiment, and the specific mechanism in vivo is required to be further investigated.
